# Evaluation of ergonomics using the DEXTER^®^ Robotic Surgery System: the ERGODEX trial

**DOI:** 10.1007/s00464-026-13251-0

**Published:** 2026-08-03

**Authors:** Dolores T. Krauss, Matilda Krones, Lisa Abry, Fabiana Castronovo, Jennifer A. Eckhoff, Stefanie Brunner, Benjamin Babic, Michael Müller, Marietta Gulz, Nico Seeger, Ryan C Broderick, Nikita Thareja, Christiane J. Bruns, Hans F. Fuchs

**Affiliations:** 1 Department for General, Visceral, Thoracic and Transplant Surgery, Faculty of Medicine and University Hospital Cologne, University of Cologne, Kerpener Str. 62, 50937 Cologne, Germany; 2 Department of Visceral and Thoracic Surgery, Cantonal Hospital of Winterthur, Winterthur, Switzerland; 3 Department of Gynecology and Obstetrics, Bern University, University of Bern, Bern, Switzerland; 4 Department of General and Visceral Surgery, Sana Klinikum Offenbach, Offenbach Am Main, Germany; 5 Division of Minimally Invasive Surgery, Department of Surgery, University of California San Diego, San Diego, CA USA

**Keywords:** Robotic surgery, Surgical ergonomics, Workflow, DEXTER Robotic Surgery System, Posture

## Abstract

**Background:**

The implications of complex robotic surgery on the well-being of surgeons remains widely unexplored. The DEXTER Robotic Surgery System offers an open concept where the surgeon console can be used in a sterile manner, allowing flexibility to switch between the robotic system and conventional laparoscopy. The aim of this study was to evaluate surgical ergonomics during the use of the DEXTER Robotic System.

**Methods:**

A prospective, non-randomized international multicenter trial evaluating surgical ergonomics during the routine clinical practice was performed in Switzerland and USA. A photo camera captured the surgeon’s posture at the console during surgery. Using picture editing programs, image analyses were created showing the surgeon’s average posture and corresponding angles. Furthermore, sound levels were measured during cases. Data were compared to previous measurements (*n* = 33 surgeries) of a closed console robotic system using the same methodology.

**Results:**

A total of 83,025 images from 40 surgical procedures were analyzed, performed by five surgeons. Knee, back, and elbow angles were within recommended ergonomic limits whereas neck and hip positions were classified as high-risk. Compared with a closed console robotic system, neck posture was significantly improved (*p* value 0.0001). Mean sound levels were 60.24 dB for all surgeries. Comparison to a robotic system with a closed console revealed a statistically quieter OR setting for the open DEXTER system (mean sound level 64.35 dB vs. 59.5 dB; *p* value 0.0001).

**Conclusion:**

The application of ergonomic measurements during a real-life setting for robotic surgery helped to reveal ergonomic advantages and challenges faced when using a new robotic technology. This is the first study providing ergonomic insights in the DEXTER surgical system, identifying individual possibilities for improvement. Future studies and recommendations will may lay the foundation for a personalized training and education in surgical ergonomics leading to a healthier workspace for all surgeons and their future careers.

**Supplementary Information:**

The online version contains supplementary material available at https://doi.org/10.1007/s00464-026-13251-0.

The introduction of laparoscopic general surgery in 1987 marked a transformative milestone in the field of surgery [1, 2]. Since then, laparoscopic and minimally invasive techniques have become the standard of care and are indispensable in daily surgical practice [3]. However, while laparoscopy is widely established, surgeons face significant ergonomic challenges, as instruments are long and mechanically inefficient relying on angulation through a fixed point of rotation [4, 5]. The introduction of robotic surgery promises to mitigate many of these ergonomic challenges, while inevitably introducing new pitfalls. Structured, scientific evaluation of ergonomics in robotic surgery remains limited in current literature, especially considering the continuously increasing number of new robotic systems being introduced to clinical practice in recent years [6, 7]. Although robotic surgery offers many advantages, including enhanced visualization, dexterity, and precision, many surgeons still prefer a laparoscopic approach due to the familiarity of the technique, cost effectiveness, and availability, paving the way for robotic systems with laparoscopic flexibility [8, 9]. The DEXTER^®^ Robotic Surgery System (Distalmotion, Epalinges, Switzerland) has recently been introduced to clinical practice in Europe and the US. The surgeon console features an open design and can be in a sterile manner and standing or sitting. It allows seamless switching between robotic and traditional laparoscopy at any time, allowing the surgeon to utilize the familiarity and expertise of a laparoscopic technique when needed while benefiting from the advantages of a robotic system [10–12]. Using a new device can increase physical and mental workload; however, combining a robotic device with familiar tools may reduce this initial strain [13, 14]. Recent investigations into surgeon ergonomics in robotic surgery have revealed significant improvement for surgeons compared to open or laparoscopic surgery. However, measurements were almost exclusively performed using the Da Vinci system (Intuitive Surgical Inc, Sunnyvale, CA, USA) [15]. A previous study has shown significant differences between posture of the surgeon when using a variety of systems, identifying the neck region as one being most influenced by ergonomic features of systems especially when comparing an open to a closed console design [16]. Considering the substantial impact of surgical ergonomics on surgeon well-being, it is high time for structured evaluation of ergonomic benefits and pitfalls of different robotic systems and scientific investigation into the practicality in real-life clinical settings. We present the first ergonomic analysis of the DEXTER Robotic Surgery System in an international, multicentric study, elaborating on factors impacting surgeons’ health today and areas for improvement in future development of robotic systems.

## Methods

**Fig. 1:**
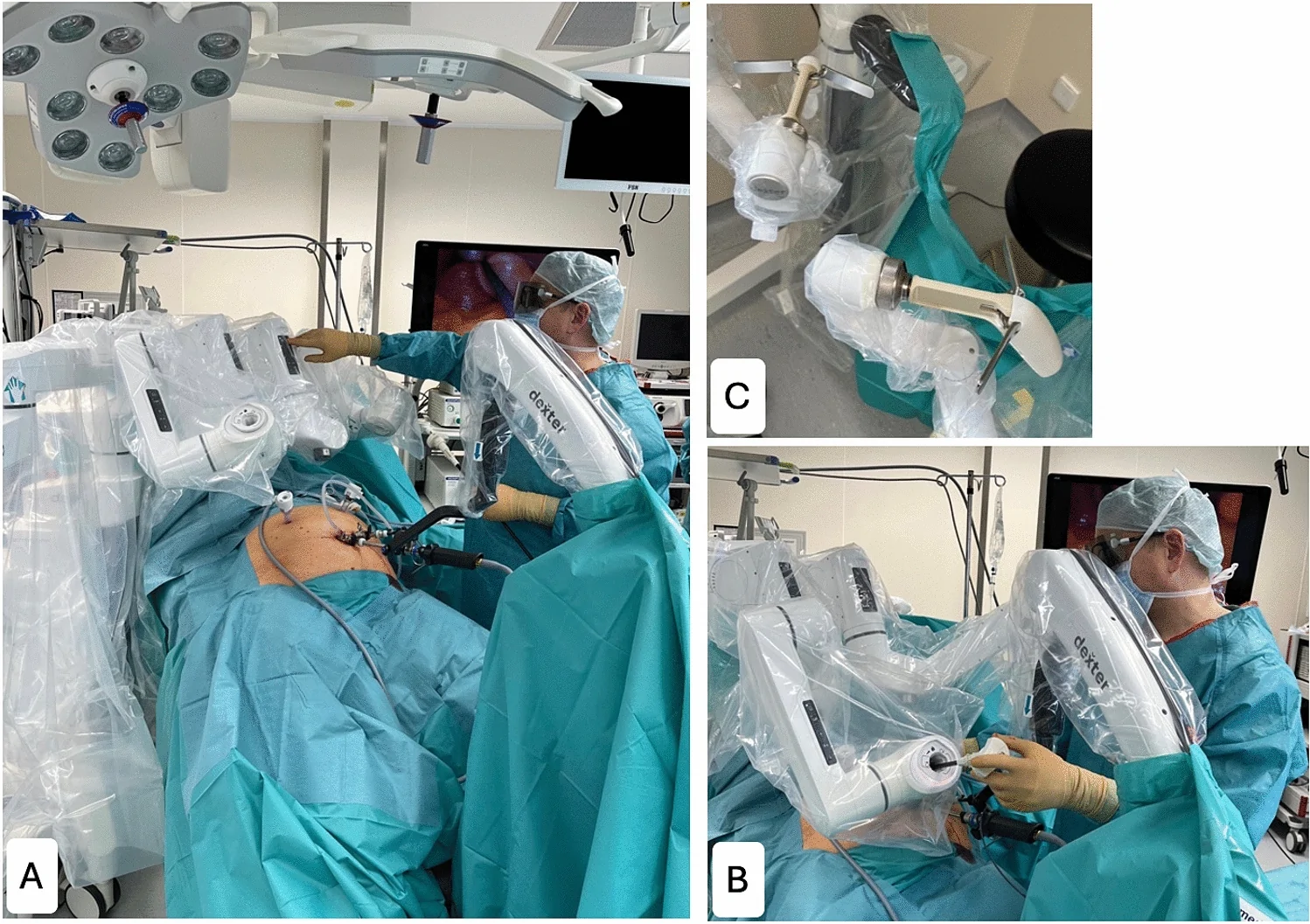
DEXTER Robotic Surgery System using an open console concept as well as a laparoscopic–robotic modular hybrid approach. **A** Robotic arms positioned in a laparoscopy mode allowing access to the surgical field. **B** Robotic arms docked and Insertion of a robotic instrument. **C** Surgeon’s console handles

A prospective, non-randomized multicenter trial evaluating surgical ergonomics during the routine use of the DEXTER Robotic Surgery System in clinical practice was performed. Study sites included the Department of Visceral and Thoracic Surgery in Winterthur, Switzerland, the Department of Gynecology in Bern, Switzerland, and the Department of Surgery of the University of California San Diego, USA. A total of 4 board-certified surgeons, who received official training in the use of the DEXTER Robotic System participated in the study. IRB approval was obtained at the University of Cologne and by all respective study sites. The DEXTER Robotic Surgery System features an open-design surgeon console that can be fully covered with sterile drapes, allowing the surgeon to remain scrubbed-in and to move quickly and freely to the patient’s bedside if desired. During the procedure, a single button press enables the robotic instrument arms to fold away into a compact laparoscopic home position without requiring system undocking, allowing certain surgical steps to be performed in laparoscopic mode (Fig. 1). Validated intraoperative measurements assessed the surgeon’s interaction with the new technology without restricting movement or affecting patient safety. All measurements entailed the use of a 3D endoscopy tower for visualization. We have previously published a detailed report of our methods for an ergonomic evaluation of robotic surgery elsewhere [16, 17]. Ergonomic evaluations of this study included posture of the console surgeon and a sound level measurement.

**Fig. 2:**
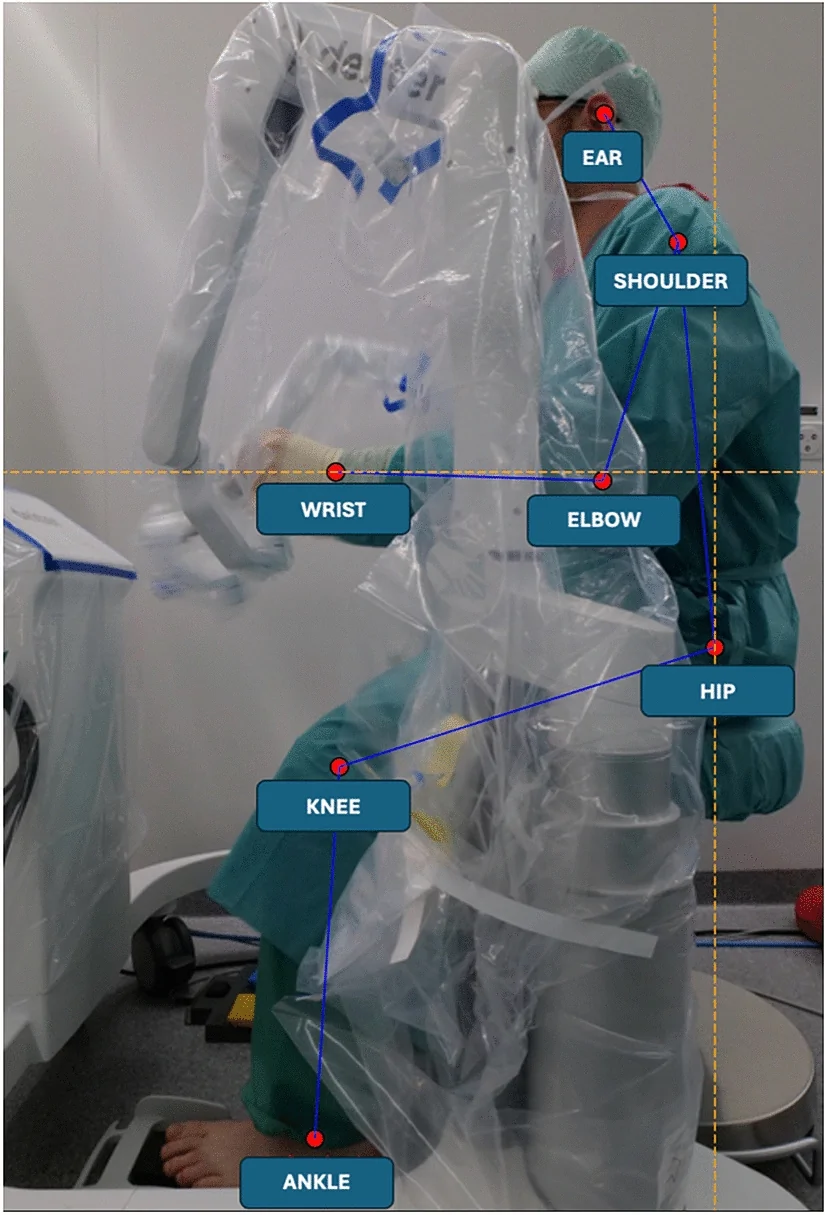
Measurement of posture during robotic assisted surgery using the DEXTER Robotic Surgery System

A photo camera was placed at a 90° angle to the sterile used console capturing the surgeon’s posture throughout the operation by taking a picture every two seconds. Images were additionally categorized to enable separate analysis of console and non-console time periods. Using picture editing programs, a merged picture was created for every 10 min of the console time showing the average posture of the surgeon. Corresponding angles were calculated, mean values obtained and compared to recommendations for a safe workplace (Fig. 2) [18–20].

Sound-level measurement was performed to evaluate the effect of an open console design on the operating theater environment as well as noise as a health hazard. The sound level was measured using the PCE-322A device (PCE Deutschland GmbH, Germany). The recorder was placed in close proximity to the console surgeon and measured solely the noise level without recording speech, maintaining data privacy. Noise levels were measured in decibels and adjusted to human hearing (dBA).

Statistical comparison and analysis were performed using SPSS Statistics (IBM, New York, USA). Mean values, standard deviations, and minimum/maximum values were reported. A subgroup analysis of ergonomics during hernia surgery compared to cholecystectomy was performed as well as a comparison of noise levels during console and non-console time. A comparison of gynecological surgery to general surgery was not performed, as the scientific value of comparing operating theater settings of two different hospitals is limited. Analysis included an unpaired *t*-test and corresponding *p*-values. *P* values of < 0.05 were considered as statistically significant.

## Results

A total of 40 surgeries using the DEXTER System were measured during this study. Of these, 15 were gynecological surgeries (i.e., hysterectomies) and 25 were general surgeries (17 hernia repairs and 8 cholecystectomies). A total of 83,025 images were obtained and analyzed, with a mean of 2076 images per surgery. Table 1 shows a summary of the posture during all measured surgeries as well as a comparison to recommendations for a safe workplace [18, 19].

**Table 1:** Evaluation of posture during a variety of surgeries using the DEXTER Robotic Surgery System

	Knee	Hip	Shoulder	Neck	Elbow	Back	Forearm
Mean (°)	102.85	99.02	28.76	21.89	117.71	5.22	5.76
Median (°)	104	98.97	27.28	22.04	115.2	5.11	5.34
Standard deviation	7.24	4.02	7.65	4.49	9.68	2	2.82
Recommendation (°)	≥ 90	110–120	N/A	10–15	≥ 90	0–10	N/A
Within recommendation	Yes	No	N/A	No	Yes	Yes	N/A

Posture was further analyzed within the variety of measured surgeries and statistical comparison was performed to compare posture during hernia surgery to cholecystectomy (Table 2). No statistical difference between posture of different general surgery procedures was seen. Table 3 summarizes obtained data per surgeon and type of surgery. 1

**Table 2:** Comparison of posture between gynecological and general surgery

Mean (°)	Gynecological surgery	Hernia surgery	Cholecystectomy	*p* value*
Knee	103.15	102.65	102.82	0.9498
Hip	96.62	99.79	100.98	0.4830
Shoulder	35.74	24.79	26.73	0.2885
Neck	20.53	22.87	21.80	0.5637
Elbow	126.86	112.74	114.48	0.4933
Back	5.39	5.51	4.36	0.2145
Forearm	7.51	5.25	4.22	0.3357

*p value comparing hernia surgery to cholecystectomy

**Table 3:** Evaluation of posture, corresponding angles, and noise levels per surgeon and type of surgery

Mean (°)	Gynecological surgery	Hernia surgery	Cholecystectomy
Surgeon 1			
*N*	0	13	5
Mean noise level (dB)	–	60.73	59.67
Knee	–	101.84	98.56
Hip	–	100.21	100.06
Shoulder	–	24.12	27.2
Neck	–	23.09	22.7
Elbow	–	112.69	116.17
Back	–	5.28	3.58
Forearm	–	5.13	3.75
Surgeon 2			
*N*	0	4	3
Mean noise level (dB)	–	59.12	59.26
Knee	–	105.28	109.92
Hip	–	98.44	102.51
Shoulder	–	26.96	25.95
Neck	–	22.16	20.31
Elbow	–	112.92	111.65
Back	–	6.26	5.67
Forearm	–	5.64	4.99
Surgeon 3			
* N*	12	0	0
Mean noise level (dB)	NA	–	–
Knee	103.59	–	–
Hip	97.71	–	–
Shoulder	38.98	–	–
Neck	20.91	–	–
Elbow	130.35	–	–
Back	4.79	–	–
Forearm	7.38	–	–
Surgeon 4			
* N*	3	0	0
Mean noise level (dB)	NA	–	–
Knee	101.81	–	–
Hip	93.37	–	–
Shoulder	26.02	–	–
Neck	19.39	–	–
Elbow	116.51	–	–
Back	7.19	–	–
Forearm	7.9	–	–

Mean sound levels were 60.24 dB for all surgeries, with a mean minimum sound level of 53.49 dB and a mean maximum sound level of 84.69 dB. No statistical difference was seen between hernia surgery and cholecystectomy (*p* value 0.6256). A separate analysis of the console versus non-console time showed a mean sound level of 59.59 dB for the console time and 59.14 dB for the non-console time with no significant difference (*p* value 0.5216).

## Discussion

The ERGODEX trial is the first multicenter trial evaluating ergonomics of the DEXTER Robotic Surgery System in a real-life setting, providing posture characteristics and comparative values for future studies, development of training curricula, and guidelines. While a variety of hospitals and procedures were measured, obtained data represent a true mean value of the ergonomic features while using the system equally representing male and female surgeons. With no difference being observed within the subgroup analysis of different procedures, obtained data represent comparable values for this analysis. Manufacturers often promote ergonomic advantages for an open console design without scientific evidence. An open console design is defined by the surgeon using an external monitor, with or without 3D glasses, allowing free-hand movement and situational awareness as well as non-verbal communication with the staff. A closed console design on the other hand is defined by the surgeon operating with their head enclosed using a fixed viewer providing an immersive and stable view but also limited direct visibility of the surgeons’ surroundings. Data from this study were further compared to previous measurements of a closed console robotic system using the same methods (ERASE Trial—Evaluation of Ergonomics and Surgeon’s Stress Levels during Robotic-Assisted Minimally Invasive Esophagectomy—submitted for publication, DaVinci Xi system). Data from this study were then compared to 33 previous measurements of a closed console robotic system using the same methods (Table 4). A statistically significant difference was observed for posture of the shoulder, neck, and elbow, with especially the neck posture improving significantly in the open console group. Comparison to a robotic system with a closed console revealed a statistically quieter OR setting for the open DEXTER system (mean sound level 64.35 vs. 59.5 dB; *p* value 0.0001).

**Table 4:** Comparison of posture between a robotic system with an open console concept and one with a closed console concept

Mean (°)	Closed console	Open console	*p* value
Knee	102.24	102.85	0.8201
Hip	99.38	99.02	0.7352
Shoulder	23.62	28.76	0.0011
Neck	33.37	21.89	0.0001
Elbow	105.05	117.71	0.0001
Back	5.37	5.22	0.8
Forearm	5.56	5.76	0.7506

Our study and this comparison were able to show a significant improvement for the neck region, which has previously been identified as an area of ergonomic high-risk posture [16, 17]. Those results are in line with a previous evaluation of another robotic system with an open console concept [17].

**Fig. 3:**
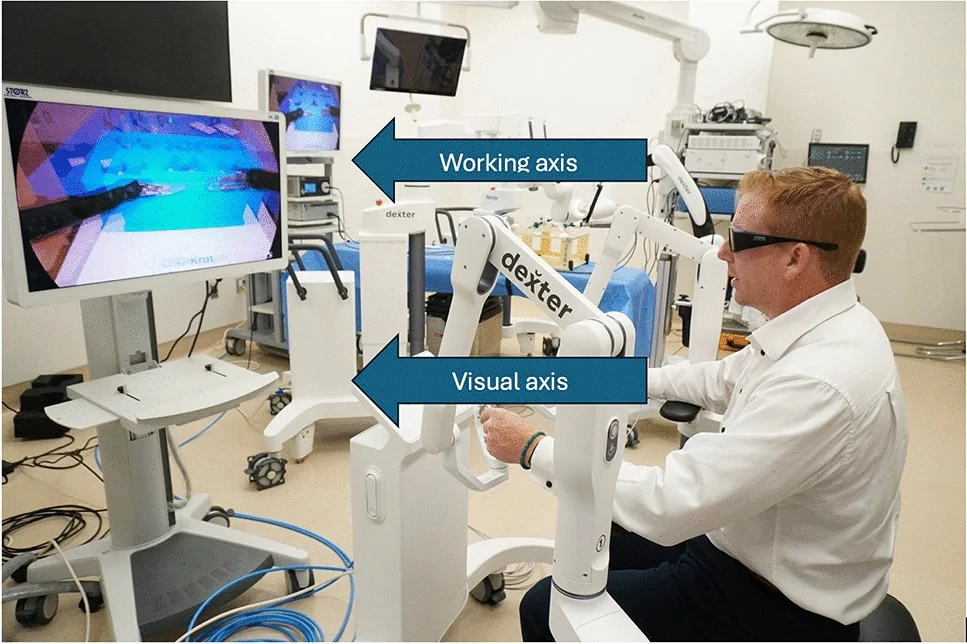
The open console of the DEXTER Robotic Surgery System enhances communication and aligns the visual and working axis of the console surgeon

Implementing a new technology, i.e., a new robotic system into clinical practice is always associated with new learning curves and a thorough evaluation of patients’ safety, while the surgeon and her/his interaction with the new system remains widely unexplored [11]. A previous study from our group has investigated posture in a variety of systems, providing an overview across systems that are currently available and in clinical practice in general surgery, identifying an urgent need for ergonomic guidelines across robotic systems [16]. Robotic consoles with a closed design rely on the user to take the time to adjust the console in the beginning of the case to maintain an ergonomically low-risk workplace throughout the procedure. However, while a system can save the console settings, making it easy in everyday practice to set up a safe workplace, surgeons often lack awareness and training in how to adjust the console correctly [21, 22]. The interaction of the surgeon with a robotic device with an open console also requires an adjustment of the console; however, the surgeon’s neck is not tied to the console, providing a rather flexible neck posture that heavily relies on the person’s individual effort to maintain a good posture. Figure 3 shows the alignment of the visual and working axis for an open console design as well as the flexibility and variability that is accompanied by an open design. In line with this, our data suggest an improvement of neck posture in an open console design, with significant differences observed in the obtained angles from ergonomic measurements.

Perception of the correct sitting posture, association with corresponding back and neck pain, and postural hypervigilance, meaning the amount of time an individual spends consciously adjusting her/his posture, are interestingly not associated with a correct posture [23]. Workers discomfort, especially in occupations with a primarily sitting workplace, has been a subject of research for many years. Posture-altering workstation interventions such as getting up, changing from sitting to standing, and break reminders have shown to not only introduce posture variability but also reduce short-term discomfort while maintaining productivity [24]. The use of the DEXTER Robotic Surgery System, which makes a switch between the operating table and the sterile used robotic console easy, can introduce this posture variability, reducing discomfort of the surgeon throughout the workday, may even enhance productivity.

The da Vinci system is the most commonly available robotic system, however, often associated with high costs making it unsuitable for smaller hospitals or less complex surgeries as reimbursement of the application of a robotic system is not yet given in many countries [25]. Modular systems such as the Dexter Robotic Surgery System provide flexibility, performing part of the surgery robotically, while other parts may be performed laparoscopically resulting in decreased cost and increase in availability as the system may be used for only parts of the surgery that are critical [26]. As hybrid systems combine the use of robotics with laparoscopic techniques, ergonomic features of both approaches must be considered when providing an evaluation of ergonomics of such a system. Ergonomics in laparoscopic surgery are mainly influenced by the use of mechanically ineffective instruments that enter the body cavity through a fixed point of rotation, relying on angulation and have been thoroughly investigated in the past. Research shows that surgeons using laparoscopic instruments exert more muscle force than while performing open surgery. The fixed entry point and required angles cause excessive flexion and ulnar deviation, especially at extreme angles (90°), resulting in musculoskeletal discomfort [27]. A study from Monfared et al. compared ergonomic data from trainees and experienced surgeons for robotic and laparoscopic procedures, showing that while robotic surgery led to lower discomfort in the upper extremities, an increased static neck and back position was found [28]. Robotic surgery has proven to lead to a reduction in localized muscle fatigue but may also be associated with an increase of stress levels of surgeons [29]. Taking this into consideration, hybrid systems may provide surgeons with the best of both worlds.

Nevertheless, while posture represents a significant part of ergonomics in the operating theater, other factors such as noise levels need to be taken into consideration. A previous study from our hospital has investigated noise level patterns in general surgery showing an average noise level 59.7 dB for laparoscopic cases, which may lead to increased stress and health hazard for patients and staff [30]. In line with these findings, sound levels measured during robotic surgery using the DEXTER System showed a mean sound level of 60.24 dB. Interestingly, no significant difference between types of surgery and console or non-console time was found. Communication is essential in the OR to conduct a successful operation and maintain patient safety, especially with the use of a robotic system, as the surgeon is physically separated from the patient, the bedside assistant, and other staff [31]. A previous study has shown that poor communication is directly linked to worse surgical outcomes, making communication a key factor in the robotic surgical setting [32]. ORs are not designed to provide great acoustics and earlier studies have shown that built-in speakers and microphones of closed console robotic devices do not solve the issue of miscommunication as disruptions in the workflow are common [33]. The DEXTER Robotic Surgery System has an open console concept with no speakers or microphones but the ability of the surgeon to communicate freely and maintain visual contact with the bedside assistant and other staff. Our findings suggest that the use of the open console does not change the sound level in the OR and therefore may lead to no disruption in the workflow as well as no impairment of communication. As part of cognitive ergonomics, a separation of the surgeon from the operating table may lead to a reduced situational awareness as well as a feeling of being detached from the case, negatively impacting the console surgeon and the cognitive workload [34]. The effects of an open console design in robotic surgery especially on cognitive workload have been previously investigated in a randomized-controlled trial using the VERSIUS system showing a significant decrease of cognitive strain for the robotic group as well as reduced ergonomic risk scores[35].

Postural awareness and hence awareness of one’s own ergonomics depicts an important factor for a long and healthy work life and can be trained. A previous study has shown that applications using artificial intelligence can significantly improve postural awareness, ultimately improve ergonomics, and reduce musculoskeletal disorders in future [36]. While an ergonomic training is included in the setup of any robotic system and manufacturers claim to adequately explain an ergonomically low-risk use of their respective systems, studies have shown that as low as 16% of surgeons using a robotic device have been previously ergonomically trained [21]. This summarizes the urgent need for international guidelines and structured training for ergonomics in robotic surgery including the variety of robotic systems on the market.

Limitations of this study include the multicenter study design as ergonomics and especially posture is often difficult to compare between multiple individual surgeons and surgeries. Anthropometric data such as surgeon’s height and arm length as well as learning curves especially prior operating hours on this specific platform are core confounding factors. These variables require adjustment in future multivariate models. Furthermore, logistics and operating room setups may vary between different hospitals, limiting the comparability of results, however, mirroring the reality of the application and everyday use of the device. Comparison of different devices may also be biased by different hospital setups, individual differences between surgeons and expertise with the respective system. Ultimately, a randomized-controlled study should include all robotic devices used for a specific field, i.e., robotic general surgery. Ergonomics would be evaluated using the same study participants (surgeons), after having completed the learning curve for the respective devices and procedures, performing the same procedure (i.e., robotic cholecystectomy). Furthermore, procedures of different complexity as well as different training levels must be measured to provide a thorough overview of ergonomics throughout the usage of the technology. However, while such a study design is not feasibly given the robotic surgical landscape at this time, data from this study and previous works will help pave the way for a clean comparison of devices in future. Using 2D kinematic imaging may, in addition limit the analysis of out-of-plane movements, creating perspective errors and bias, while being time efficient and an accurate analysis for planar movements such as sitting at the console. Incorporating additional methods such as surface electromyography (sEMG), the RULA (rapid upper limb assessment) score, or motion capture systems, i.e., would help capture isometric contraction fatigue supplementing other aspects of posture and should be incooperated into future studies. Posture measurements were further limited by only evaluating the console part of the surgery, while no posture evaluation was performed for the non-console laparoscopic time. Thorough evaluations of ergonomics during laparoscopic surgery, however, have been performed in the past, filling this gap sufficiently. Future studies need to focus on evaluating not only posture and noise but also other factors that may influence the interaction of the surgeon with a new device. Data must be used to create, evaluate, and implement a structured training curriculum for ergonomics across systems as the standard for robotic trainees in everyday practice.

## Conclusion

The application of ergonomic measurements during a real-life setting for robotic surgery helped to reveal ergonomic advantages and challenges faced when using a new robotic technology. This is the first study providing ergonomic insights in the DEXTER surgical system, identifying individual possibilities for improvement. Future studies and recommendations will may lay the foundation for a personalized training and education in surgical ergonomics leading to a healthier workspace for all surgeons and their future careers.

## Supplementary Information

Below is the link to the electronic supplementary material.

Supplementary file1 (JPG 458 KB)
